# Case Report: Ventricular preexcitation-induced dilated cardiomyopathy improved by the pharmacologic suppression of ventricular preexcitation in three infants, and literature review

**DOI:** 10.3389/fped.2024.1302534

**Published:** 2024-03-01

**Authors:** Dai Chencheng, Zhong Min, Shi Wanming, Shangguan Wen, Guo Baojing, Xiao Yanyan, Han Ling, Long Deyong

**Affiliations:** ^1^ Department of Pediatric Cardiology, Capital Medical University Affiliated Beijing Anzhen Hospital, Beijing, China; ^2^ Department of Ultrasound, Meizhou People’s Hospital, Guangdong, China; ^3^ Department of Cardiology, Capital Medical University Affiliated Beijing Anzhen Hospital, Beijing, China

**Keywords:** ventricular preexcitation, dilated cardiomyopathy, left ventricular dyssynchrony, amiodarone, propafenone

## Abstract

The therapy of ventricular preexcitation-induced dilated cardiomyopathy in very small infants or infants with a high risk of ablation is tough and related articles are rare. Effective pharmacotherapy to suppress ventricular preexcitation is valuable.

**Aims:**

To evaluate the effectiveness and safety of pharmacotherapy for cardiac resynchronization in infants with ventricular preexcitation-induced dilated cardiomyopathy.

**Methods and results:**

Three infants with ventricular preexcitation-induced dilated cardiomyopathy, due to the disappearance of ventricular preexcitation during the placement of catheter, intermittent WPW pattern, and right mid septal accessory pathway respectively, had received pharmacotherapy for cardiac resynchronization. The initial dosage of oral amiodarone was 5 mg/kg.d and it was followed by the maintenance dosage of 2–2.5 mg/kg.d 4 weeks later. Propafenone (15 mg/kg.d) served as a supplement since amiodarone was not adequate in case 3. The three infants achieved successful pharmacologic suppression of ventricular preexcitation 10, 6.5, and 4.5 weeks after the initiation of amiodarone respectively. They all got normalized contraction of interventricular septum and LVEF as well as reduced LVEDD gradually after the disappearance of ventricular preexcitation. No side effects associated with pharmacotherapy happened during the follow-up. Amiodarone had been withdrawn for 2 years and 5 months in Cases 1 and 2. They both remained free from ventricular preexcitation and retained normal LVEF and LVEDD.

**Conclusions:**

Pharmacotherapy for cardiac resynchronization with oral amiodarone or in combination with propafenone for infants with ventricular preexcitation-induced dilated cardiomyopathy is effective and safe. Pharmacotherapy for cardiac resynchronization served as another therapeutic choice besides ablation.

## Introduction

Type B ventricular preexcitation may cause abnormal interventricular septal motion and left ventricular (LV) dyssynchrony in patients with WPW pattern/syndrome, which can induce LV dysfunction and dilation. Some patients even develop ventricular preexcitation-induced dilated cardiomyopathy (DCM) (1–8). With more and more related cases being reported worldwide, ventricular preexcitation with ventricular dysfunction presumed due to dyssynchrony in larger patients (weight ≥ 15 kg) or when medical therapy is either not effective or associated with intolerable adverse effects in smaller patients had been listed as ablation indications (class IIa) (9, 10). However, for infants weighing less than 7 kg, ablations are hard to perform due to the small diameter of the femoral veins especially in the areas without special ablation catheters designed for pediatric patients. Medical treatments with anti-heart failure drugs do not respond well in most cases. The application of digoxin is limited due to ventricular preexcitation, which proposes another obstacle to the therapy.

Cardiac resynchronization aimed to suppress anterograde conduction of accessory pathways (APs) by effective pharmacotherapy is quite valuable in low-body-weight infants and infants with anteroseptal/mid septal APs which bring a high risk of Ⅲ°atrioventricular block secondary to ablation or patients who can not be ablated successfully. Related articles are quite limited. We have treated three cases of ventricular preexcitation-induced DCM with different situations successfully by the suppression of ventricular preexcitation through amiodarone or in combination with propafenone.

## Methods

### Patients

**Figure 1:**
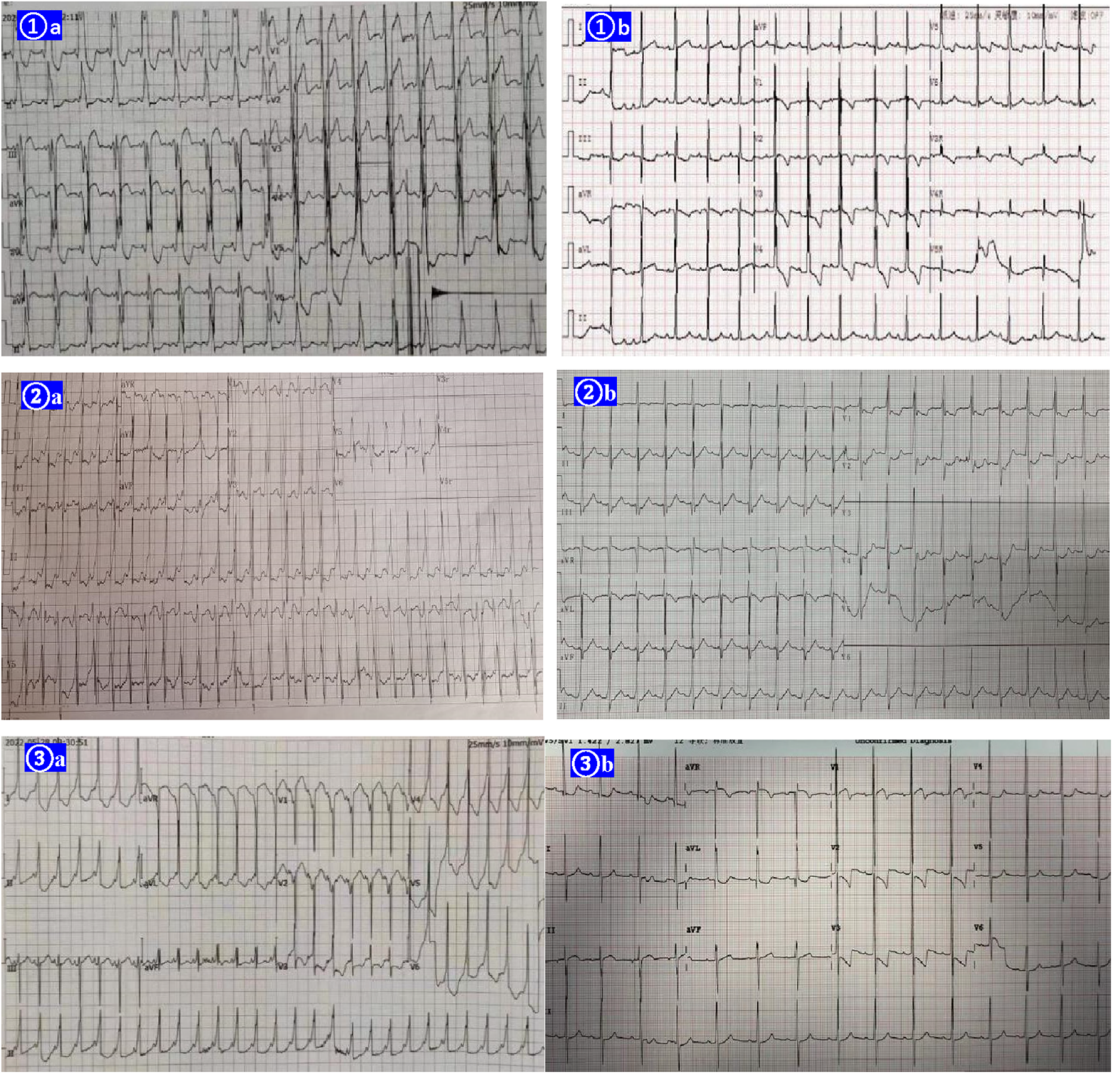
EKGs of the three cases before the therapy of amiodarone shown right postlateral, lateral, and midseptal manifest accessory pathways respectively (①**A** ②**A** ③**A**). Amiodarone alone (cases 1 and 2) or in combination with propafenone (case 3) suppressed the ventricular preexcitation successfully in the 3 cases (①**B** ②**B** ③**B**).

Three consecutive female infants with DCM and WPW pattern/syndrome, who underwent oral pharmacotherapy meditation to suppress ventricular preexcitation through amiodarone or combination with propafenone besides anti-heart failure drugs between July 2020 and May 2022, were included in this study. The vectors of the delta waves of the three cases suggested right anterolateral, posterolateral, and midseptal pathways (Figure 1). DCM was defined as a literature description (11). All the infants presented with the clinical signs and symptoms of chronic congestive heart failure, such as fatigue and exertional dyspnea, and they were at the low end of physical growth compared with infants in the same age group. Cases 1 and 2 never complained of tachycardia-related symptoms. Periodical Holters (ECG Holter's recordings) were performed on the two patients and showed no tachycardia episodes. Case 3 was admitted to the local hospital for paroxysmal supraventricular tachycardia (PSVT). After termination of it, continuous ECG monitoring for 1 month did not show the onset of PSVT. However, the left ventricular systolic function did not improve with the remission of PSVT. Incessant tachycardia as the cause of DCM was not considered. All patients underwent routine and comprehensive diagnostic screening for the common etiologies of DCM and the results were negative for all patients. They were treated with routine anti-heart failure chemotherapy. The dosage of drugs was not altered after the administration of amiodarone until the patients achieved recovery of their LV function. The Declaration of Helsinki was reflected in *a priori* approval by the institution's human research committee, and it was approved by the Ethics Committee of Capital Medical University affiliated with Beijing Anzhen Hospital.

### Electrocardiography and echocardiography

Twelve-lead electrocardiography during sinus rhythm was conducted in all patients before and after the administration of pharmacotherapy. Transthoracic echocardiographies were performed with an iq echo system (GE Medical Systems). All patients underwent echocardiographic examinations before and 1, 3, 6, 12, 18, and 24 months after the pharmacotherapy. Their global LV function was assessed by measuring end-diastolic and end-systolic LV diameters from a parasternal long-axis M-mode echocardiogram. The left ventricular ejection fraction (LVEF) was calculated using the biplane Simpson's method. The basal interventricular septal thickness was measured from the apical 4-chamber view at the end-systole. Intraventricular dyssynchrony through M-mode echocardiography was quantified as the time delay between the peak septal systolic motion and the left posterior wall systolic motion (septal-to-posterior wall motion delay, SPWMD) (12). Speckle tracking echocardiography was applied for the evaluation of LV dyssynchrony. The two-dimensional LV longitudinal strain was analyzed through the apical 4-, 2- and 3- chamber long-axis views (13, 14). The standard deviation of the time to the peak systolic strain (Ts-SD) over 18 longitudinal segments was measured. Echocardiography was performed by the same physician and was analyzed online.

### Pharmacotherapy for cardiac resynchronization

We planned to perform ablation for case 1 with propofol as the anesthetic. When placing the coronary sinus electrode, the delta wave disappeared abruptly. Ventricular pacing showed no retrograde conduction of the AP. Atrial pacing in several sites of the right atrium did not show antegrade conduction through it. The delta wave did not recover 1 h after the anesthetic was reduced and all the electrodes were removed. Two hours after returning back to the ward, persistent ventricular preexcitation appeared. Case 2 had intermittent ventricular preexcitation and the proportion of preexcitation was about 60%. Case 3 had a midseptal AP indicated by the EKG. The initial dosage of oral amiodarone was 5 mg/kg.d and the maintenance dosage of 2–2.5 mg/kg.d 4 weeks later. Case 2 encountered an episode of PSVT for about 1 h which was terminated by intravenous propafenone when she had been treated by amiodarone for 23 days. Her LV dysfunction did not deteriorate due to the second onset. After that, oral propafenone (15 mg/kg.d) was added. PSVT did not recur from then on.

## Results

**Figure 2:**
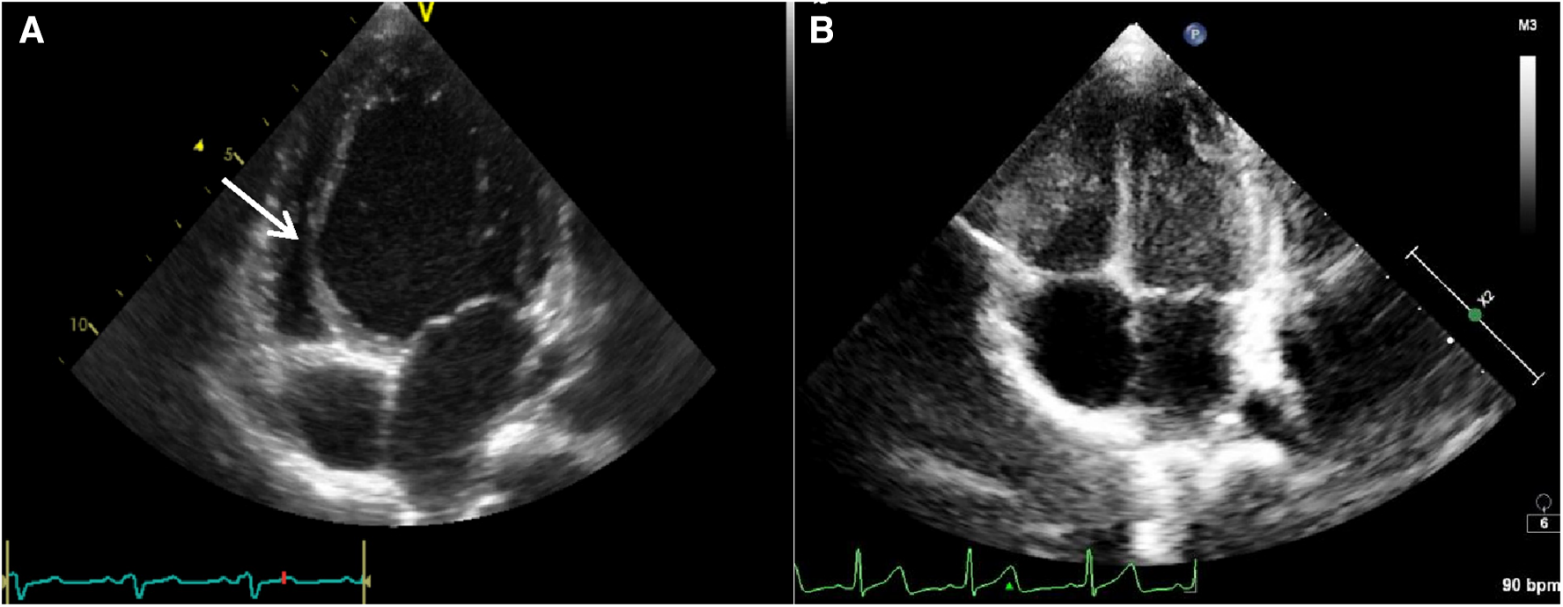
The parasternal long axis four-chamber view of case 1 showed the left ventricle was spherically dilated and the basal/middle segment of the interventricular septum (IVS) contracted paradoxically like an aneurysm (see the arrow) protruding into the compressed right ventricle before pharmacotherapy for cardiac resynchronization (**A**). The paradoxical motion of the interventricular septum disappeared and the diameter of LV returned to normal after successful pharmacotherapy for cardiac resynchronization (**B**).

**Figure 3:**
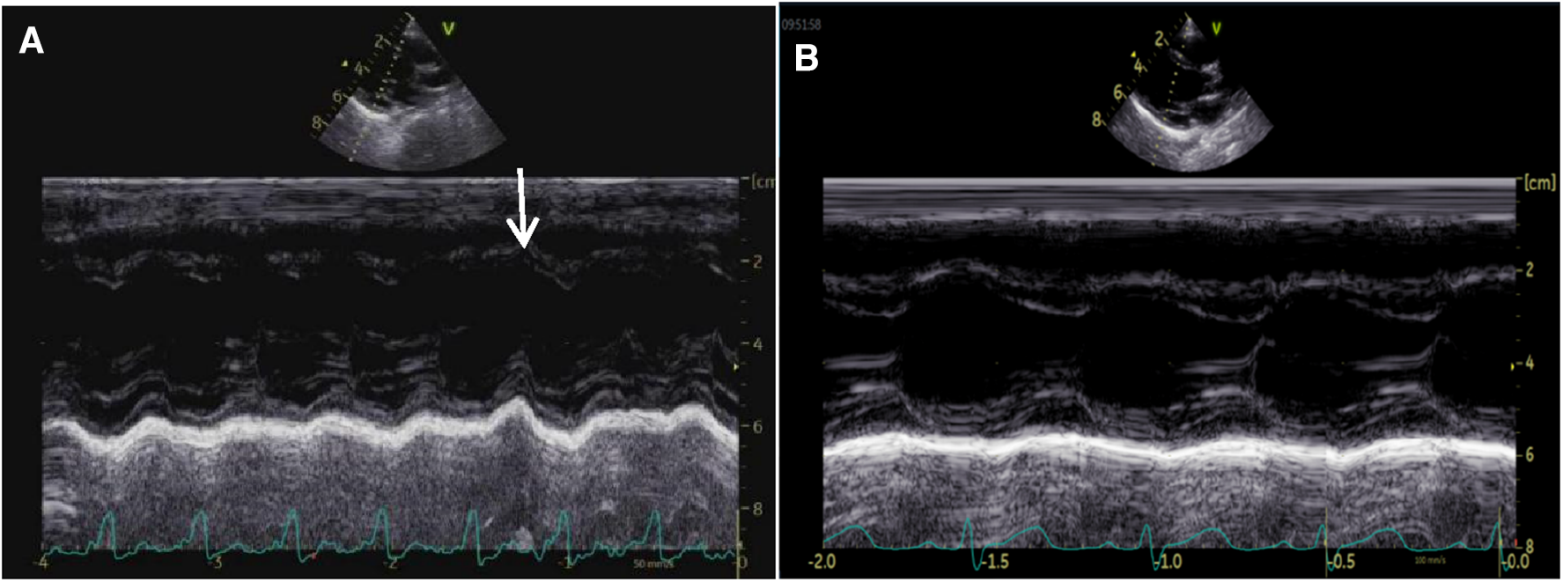
M-mode tracing from a parasternal long-axis view of case 2 before the therapy of amiodarone, showing interventricular septal paradoxical motion (**A**, arrow). The ventricular septum and LV post wall moved normally 6 months after the therapy of amiodarone (**B**).

**Figure 4:**
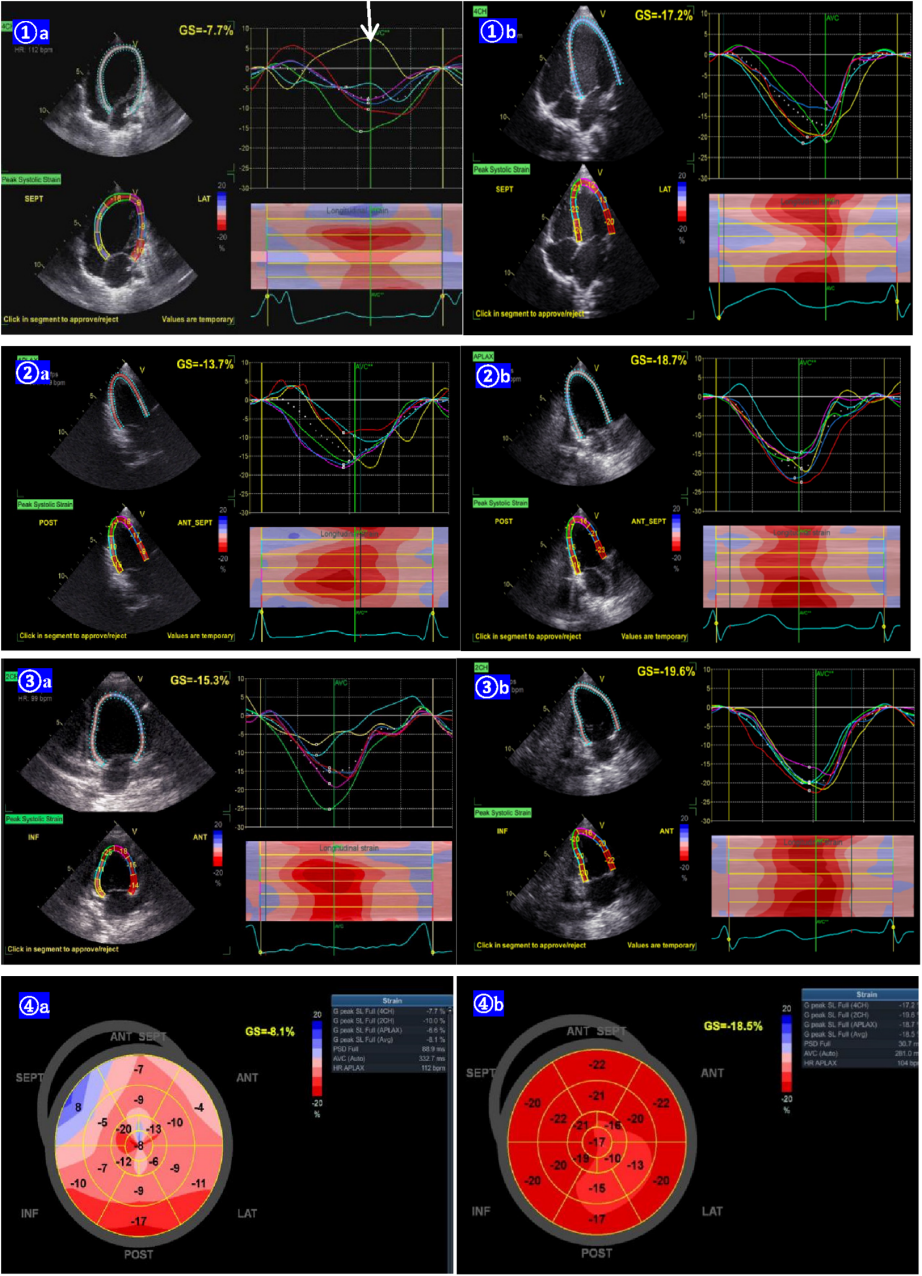
Lv longitudinal strain curves from the apical 4-chamber (1**A**), 2-chamber (2**A**), and 3-chamber (3**A**) view of case 2 before the pharmacotherapy of amiodarone, showing the basal segments of the interventricular septal paradoxical motion (arrow, positive strain). The LV longitudinal strain becomes normal 6 months post pharmacotherapy (1**B**,2**B**,3**B**). The bull's eye of the LV longitudinal strain before the pharmacotherapy, shows the segment of the post-interventricular septal wall paradoxical motion (positive strain). The bull's eye of the LV longitudinal strain changed to normal 6 months after the pharmacotherapy (4**B**).

The clinical characteristics and echocardiographic investigations of the three infants are shown in Supplementary Table S1. The abnormal interventricular septal motion presented as obvious rightward systolic bulging (Figure 2A). The basal/middle segments of the interventricular septum moved similar to an aneurysm, with typical bulging during end-systole. The M-mode echocardiogram showed paradoxical motion between the interventricular septum and posterior wall of the LV (Figure 3A). The interventricular septal paradoxical motion of the basal segments was shown by the longitudinal strain analysis, with positive strains that were opposite to the other segments (Figure 4A).

The three infants achieved successful pharmacologic suppression of ventricular preexcitation using amiodarone or combination with propafenone 10, 6.5, and 4.5 weeks after the initiation of amiodarone respectively. Case 1 started to show intermittent ventricular preexcitation and the proportion of preexcitation was about 50% at the week 10, followed by her LVEF increasing gradually. Holter scans showed ventricular preexcitation disappeared completely at week 18. After 22 weeks of treatment, echocardiography showed that paradoxical motion of the IVS had disappeared. The LVEF and LVEDD completely returned to normal with cardiac resynchronization (Figure 2B, Supplementary Table S1). Cases 2 and 3 also experienced remarkable improvement in LVEF and reduced LVEDD following the disappearance of ventricular preexcitation (Supplementary Table S1). All three infants had normal thyroid function and no abnormalities were found in eyes and lungs during the follow-up. Amiodarone had been withdrawn for 2 years and 5 months in cases 1 and 2. Ventricular preexcitation did not recur in the 2 cases; they obtained normal growth and development and continued to retain normal LVEF and LVEDD.

## Discussion

The development of ventricular preexcitation-induced dilated cardiomyopathy was heterogeneous. Some children still had better compensatory cardiac function in adolescence. However, infants tended to show obvious decompensated cardiac insufficiency. Previous studies have confirmed that ablation can reverse the disease (1–8, 11, 12). We reported ventricular preexcitation-induced dilated cardiomyopathy in China in 2013 firstly and have accumulated lots of experiences in ablation (1, 7, 8). However, ablation is not always preferable for every infant, especially those with low body weight, high risk, or difficulty in ablation. Pharmacotherapy for cardiac resynchronization may serve as a supplement for ablation in some instances.

Our first case had a proper indication for ablation since anti-heart failure drugs were ineffective. However, her ventricular preexcitation disappeared suddenly during the process. Ablation could not be performed since the AP had no retrograde conduction and antegrade conduction could not be provoked. So, pharmacologic suppression of ventricular preexcitation was tried. Her left ventricular function and diameter became completely normalized following the successful suppression of ventricular preexcitation. Even after the discontinuation of amiodarone, she maintained normal heart function and was free from ventricular preexcitation. Amiodarone was also prescribed for intermittent ventricular preexcitation and mid septal AP in the following 2 cases and also gained satisfactory effects. Treated with the dosage of 5 mg/kg.d, case 3 still suffered from an episode of PSVT. Propafenone was added to control the onset of PSVT. Due to the combined effect, it just took 4.5 weeks for successful suppression of ventricular preexcitation.

Reports associated with pharmacologic therapy for cardiac resynchronization in infants with ventricular preexcitation-induced DCM were rare. There have been just seven cases reported (15–20) (Supplementary Table S2). Five cases used amiodarone and one case failed. One case chose propafenone and another chose flecainide. Cadrin Tourigny (15) reported that two infants with severe ventricular preexcitation-induced DCM were successfully treated with amiodarone to suppress the antegrade conduction of the AP, which obviated their need for heart transplantation. One case remained free from ventricular preexcitation and heart failure 10 years after withdrawal of amiodarone. Further ablation was still needed in another case. Suzuki Y (16) reported amiodarone was chosen for cardiac resynchronization after twice unsuccessful catheter ablations in a 6-month-old infant weighed 6.8 kg and attained satisfactory effect. However, amiodarone was not always effective (5). Besides amiodarone, flecainide and propafenone were also reported to be effective in such cases (17, 18). Since amiodarone had more positive evidence, we chose it as a preference. Long-term administration of amiodarone-related adverse effects such as interstitial lung disease, abnormal thyroid function, and corneal pigmentation should be paid attention to. In order to avoid the adverse effects, our maintenance dosage was 2–2.5 mg/kg.d.

Although it has been reported that catheter ablation may be a safe and effective treatment option in right free wall accessory pathways (21), catheter ablation for any reason is infrequently performed in infants less than 6 months of age considering the increased risks associated with the procedure. Of course, if small-size ablation catheters designed for children are available, catheter ablation can also be considered the first choice in centers with ample experience. Moreover, for infants with low body weight or anteroseptal/midseptal APs with a high risk of Ⅲ°atrioventricular block secondary to ablation, pharmacologic suppression of ventricular preexcitation is more sensible compared with ablation. In addition, the natural evolution of accessory pathways in infants, with a 36% rate of spontaneous resolution, favors a less aggressive approach (22). If antegrade conduction of the AP can not resolve spontaneously, amiodarone may postpone the need for ablation, so that the infants can grow and develop with normal cardiac function, and avoid the risks of ablation due to the low body weight.

## Conclusions

Pharmacotherapy for cardiac resynchronization with oral amiodarone or in combination with propafenone for infants with ventricular preexcitation-induced dilated cardiomyopathy is effective and safe. Pharmacotherapy served as another therapeutic choice besides ablation for infants with low body weight, anteroseptal/midseptal APs, or intermittent ventricular preexcitation.

## Data Availability

The original contributions presented in the study are included in the article/**Supplementary Material**, further inquiries can be directed to the corresponding author/s.
